# Circularly polarized luminescence from Tb(iii) interacting with chiral polyether macrocycles†‡

**DOI:** 10.1039/d2dt02627a

**Published:** 2022-10-04

**Authors:** Alexandre Homberg, Federica Navazio, Antoine Le Tellier, Francesco Zinna, Alexandre Fürstenberg, Céline Besnard, Lorenzo Di Bari, Jérôme Lacour

**Affiliations:** Department of Organic Chemistry, University of Geneva Quai Ernest Ansermet 30 1211 Geneva 4 Switzerland Jerome.lacour@unige.ch; School of Science and Technology, Chemistry Division, University of Camerino via S. Agostino n. 1 62032 Camerino Italy; Dipartimento di Chimica e Chimica Industriale, Università di Pisa Via Moruzzi 13 56124 Pisa Italy; Department of Inorganic and Analytical Chemistry, University of Geneva 1211 Geneva Switzerland; Department of Physical Chemistry, University of Geneva 1211 Geneva Switzerland; Laboratory of Crystallography, University of Geneva Quai Ernest Ansermet 24 1211 Geneva 4 Switzerland

## Abstract

A straightforward two-step synthesis protocol affords a series of chiral amide-based bis-pyridine substituted polyether macrocycles. One ligand is particularly able to complex terbium(iii) ions spontaneously. Upon complexation, interesting chiroptical properties are observed both in absorbance (ECD) and in fluorescence (CPL). In ligand-centered electronic circular dichroism, a sign inversion coupled with a signal enhancement is measured; while an easily detectable metal-centered circularly polarized luminescence with a *g*_lum_ of 0.05 is obtained for the main ^5^D_4_ → ^7^F_5_ terbium transition. The coordination mode and structure of the complex was studied using different analysis methods (NMR analysis, spectrophotometric titration and solid-state elucidation).

## Abstract 2

Chiral amide-based bis-pyridine polyether macrocycles interact with lanthanides. In the case of luminescent Tb(iii), the host-guest interaction can be observed by the ligand-centered ECD and by Tb-centered CPL.

## Graphical abstract

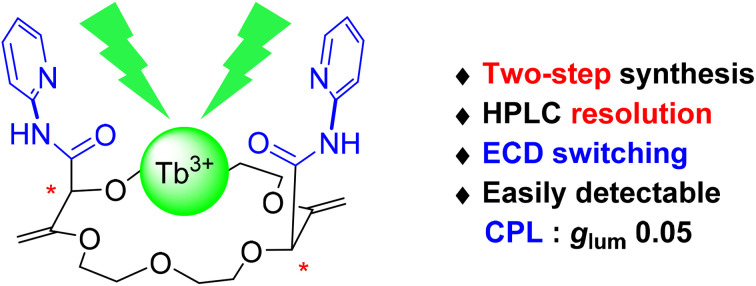

## Introduction

**Fig. 1:**
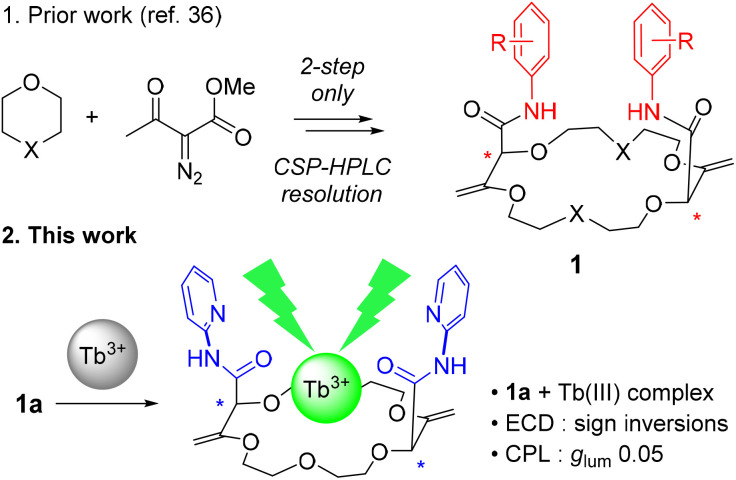
Synthesis and properties of chiral lanthanide complexes.

**Fig. 2:**
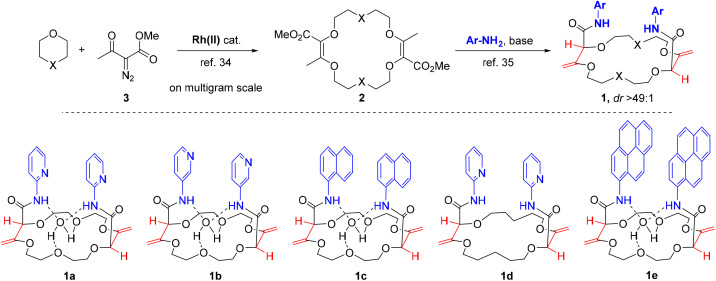
Two-step synthesis of the ligands of the study.

The study of chiral lanthanide complexes is a strong and developing field of research.^1–26^ These complexes often display interesting circularly polarized luminescence (CPL) properties and,^27^ for this reason, applications can be found in key research areas such as in bioanalytical assays and imaging^28–39^ or circularly polarized OLEDs.^40–44^ To promote efficient chiroptical properties, polydentate monoligands, such as triazacyclononane or tetraazacyclododecane, are usually employed.^2^ However, fully oxygenated ligands remain scarce in the design of chiral lanthanide complexes,^45^ possibly due to limited examples of efficient syntheses of such chiral functionalized compounds. Recently, the direct synthesis of a new family of highly functionalized chiral polyether macrocycles of type 1, made in only two steps from cyclic ethers, was reported.^46–48^ These molecules find applications in a variety of fields^49,50^ from analytical^51^ and supramolecular^52^ sciences to catalysis,^53^ luminescence^54–57^ and photophysics.^58–60^ Herein, in a new development, we report that the complex formed by ligand 1a (X = O, Fig. 1 and 2) and terbium(iii) triflate display strong electronic circular dichroism (ECD) but also CPL properties. Upon Tb complexation, in ECD, transitions sign inversion and signal enhancement are observed while in CPL a *g*_lum_ of 0.05 is measured for the main ^5^D_4_ → ^7^F_5_ terbium transition. In addition, ^1^H-NMR analysis, spectrophotometric titrations and solid-state elucidation of parent complexes were performed to confirm the proposed binding mode of the metal ion and the ligand macrocycle.

## Results and discussion

### Two-step straightforward synthesis of ligands

Practically, ligands of type 1 were synthetized in two steps from 1,4-dioxane or tetrahydropyran (THP) (Fig. 2). First, unsaturated macrocycles 2 were synthesized by [3 + 6 + 3 + 6] Rh(ii)-catalyzed condensations of 1,4-dioxane or THP with α-diazo-β-ketoester 3 on multigram scale (*e.g.*, 72% for X = O).^47,61,62^ Then, with compounds 2 in hand (X = O or CH_2_) and using α and β amino-pyridines or 1-naphthylamine as nucleophiles, a series of chiral macrocycles was prepared following a double tandem [amidation + olefin transposition] protocol to afford bisamides 1a to 1d as single diastereoisomers (Fig. 2, 43–60%, dr > 49 : 1).^48^

For each compound, the enantiomers could be efficiently separated by chiral stationary phase (CSP) HPLC on a semi-preparative scale using CHIRALPAK® IG column and a mixture of CH_2_Cl_2_–MeOH (99 : 1, 0.1% diethanolamine) as mobile phase (see ESI† for details).^56^

### Optical properties of the terbium complex

**Fig. 3:**
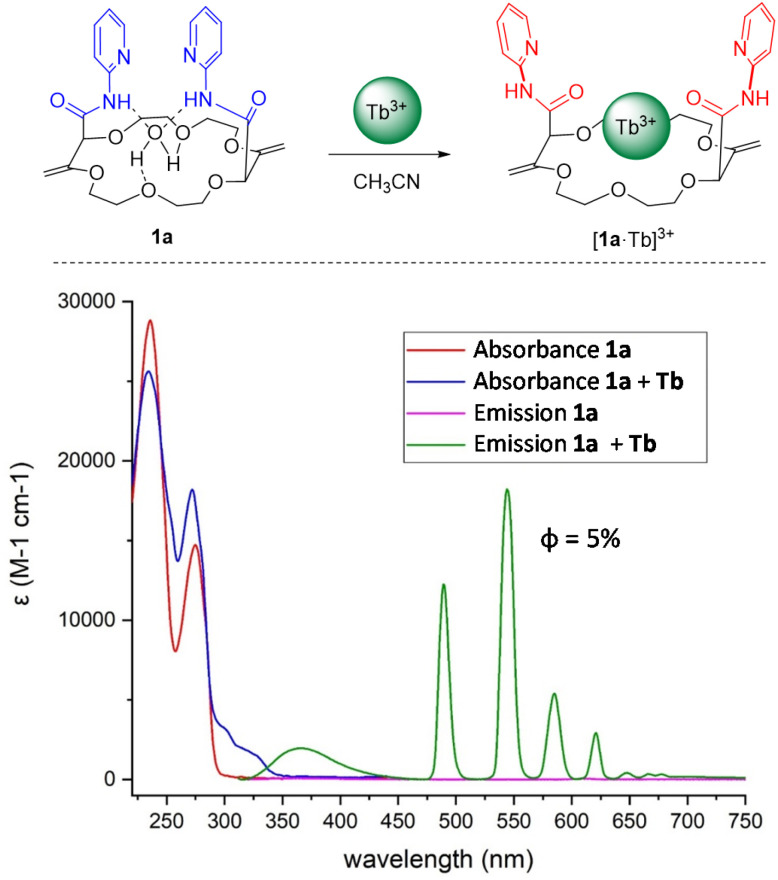
Top: Formation and structure of the metal complex. Bottom: Absorbance (red and blue lines) and fluorescence (pink and green lines) spectra of ligand 1a without (red and pink lines) or with 3.0 equivalents of Tb(iii) (blue and green lines). Quantum yield relative to 9,10-diphenylanthracene (solvent: acetonitrile, concentration: *ca.* 0.6 × 10^−6^ M, *λ*_ex_: 305 nm).

With the four candidates 1a–1d in hand, the ability of these macrocycles to act as antennas for lanthanide emission (ligand-to-terbium energy transfer) was assessed qualitatively. Of the four moieties, 2-pyridine substituted 1a exhibited, in the presence of an excess of terbium(iii) triflate and upon ligand excitation (366 nm), the most intense characteristic green emission (see ESI† for details). Consequently, the absorption and emission spectra of 1a and of a mixture of 1a with 3.0 equivalents of Tb(iii) were recorded in acetonitrile (Fig. 3, bottom). The four characteristic major and sharp emission bands of the ^5^D_4_ → ^7^F_*j*_ (*j* = 6 − 3) terbium transitions were observed at 485 nm, 545 nm, 580 nm and 620 nm respectively with a total quantum yield of 5% (relative to 9,10-diphenylanthracene).

The formation of the complex between 1a and terbium(iii) was then monitored through spectrophotometric titration, firstly using UV-Vis absorption spectroscopy. In the titration experiment, aliquots of a Tb(OTf)_3_ solution were added to a macrocycle solution (*ca.* 0.5 × 10^−5^ M) in acetonitrile. The free ligand spectrum (red line) displays two strong absorption bands with maxima at 238 nm and 270 nm which can be assigned to the π → π* transitions of pyridine. Upon Tb(OTf)_3_ addition, the two main absorption bands behave differently: the most red-shifted one displayed a small hyperchromic effect while the more blue-shifted experienced a small hypochromic effect. Furthermore, as the equivalents of terbium(iii) increased, a new weak band around 300 nm appeared signaling the coordination of the lanthanide with macrocycle. Above 1.5 equivalents of terbium salt, no further evolution of the spectra was observed (Fig. S6†). To better understand the coordination behaviour of terbium inside the ligand, an analogous titration of 1a was performed with Ba(ClO_4_)_2_, a salt for which a 1 : 1 stoichiometry and coordination geometry have been already previously established with related macrocycles.^55,56^ The results are reported in Fig. S8† in which the two main transitions evolve, similarly to the terbium titration, with the related hypo-hyperchromic effect. This indicates analogous binding modes for the two metal salts and hence a 1 : 1 complexation between the terbium and macrocycle 1a. More precisely, we propose that the terbium replaces the coordinated water molecule. The coordination of the metal occurs with the six oxygen atoms of the polyether ring while the two carbonyl groups of the amides rotate inward to reach an eightfold coordination pattern (Fig. 3, top). The interaction of 1a with terbium(iii) is clearly appreciated in emission. The luminescence spectrum displays the characteristic sharp bands associated to f–f Tb(iii) transitions, which reach an intensity maximum upon the addition of 1.5 equivalents of lanthanide.^63^ Also, the luminescence lifetime of complex [1a·Tb][ClO_4_]_3_ at 545 nm at a concentration of 0.1 mM upon excitation at 305 nm was determined to be 1.9 ± 0.1 ms both in acetonitrile and acetonitrile-d_3_ solutions (Fig. S17†). This finding indicates that either no labile solvent molecule is present in the first coordination sphere of Tb(iii) in the complex or that C–H vibrations in acetonitrile do not lead to efficient deactivation of the excited complex. The lifetime of Tb(iii) in dry acetonitrile was reported to be 1.87 ms for Tb(NO_3_)_3_, similar to the lifetime of complex [1a·Tb][ClO_4_]_3_, and 2.42 ms for Tb(ClO_4_)_3_.^64^ It seems therefore highly unlikely that a water molecule is present in the first coordination sphere of the complex in acetonitrile, unless this water molecule cannot be exchanged for an acetonitrile molecule in solution. The solubility of 1a in water was too low to be able to induce complex formation in aqueous solution, otherwise the lifetime of Tb(iii) would be significantly shorter.^65,66^

### Coordination mode of the terbium complexes

To rationalize the emission (chir)optical properties, care was taken to demonstrate that terbium coordination occurs within the macrocycle ring (and not with the pyridine moieties). Changing the antennas to regioisomeric aminopyridines (ligand 1b), or different extended aromatic moieties (1c) gave similar results in binding studies. Ligands 1b and 1c also allowed ligand-to-terbium energy transfer, resulting in the characteristic green emission (Fig. S9 and S10†). However, with ligand 1d, which lacks two oxygen atoms in the macrocyclic platform in comparison to 1a, only a very weak emission pattern of Tb^3+^ was observed (Fig. S11†).

This corroborates the hypothesis that the terbium ion coordinates to the polyether ring and requires six ether and two carbonyl oxygen atoms to complete its coordination sphere. However, to ascertain this coordination mode, further analyses were performed to determine more precisely the nature of the lanthanide ion/macrocycle interaction. First, ^1^H-NMR spectroscopic analyses were carried out. For the complexation study, diamagnetic lutetium(iii) cation was selected as the paramagnetic character of the Tb(iii) does not permit direct analysis in the present case. Spectra were recorded in the presence of different equivalents of LuCl_3_ (0, 0.5, 1 and 2 equivalents) with ligand 1a in CD_3_CN (*c* = 15 mM, 600 MHz, 298 K, Fig. S15†). Upon titration, the following was noted: (i) only a broadening of the aromatic protons signal occurs while the amide protons are shifted downfield (Δ*δ* = +0.8 ppm); (ii) the remainder of the signals, for instance of the polyether ring, undergoes negligible changes after Lu(iii) addition; (iii) the structure conserves a local symmetry, most probably *C*_2_. This data, and the deshielding of the N–H signals particularly, corroborates the postulated rotation of the amide moieties to provide two carbonyl groups for metal binding. Also, on the NMR time scale, only one global set of signals are observed upon titration, which tends to indicate a fast host–guest exchange.^8^ Such a kinetic lability of the lanthanide ion might be a reason for the difficulty experienced in the attempts to isolate the targeted metal complex.

**Fig. 4:**
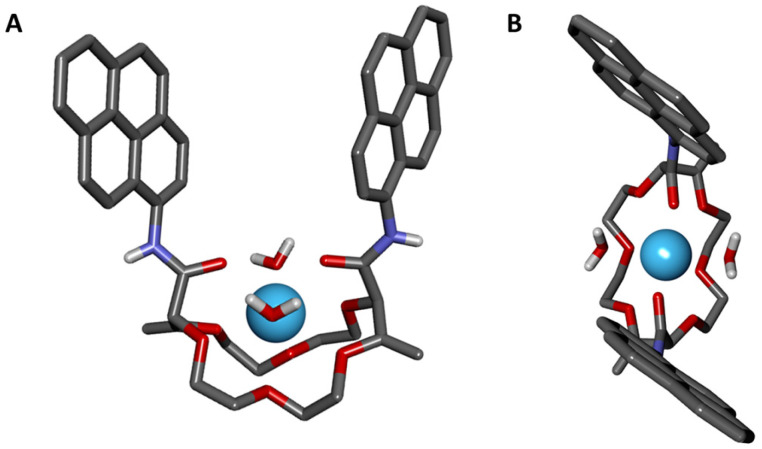
Stick view of the crystal structure of [1e·La·(H_2_O)_2_]^3+^ (A: side view; B: top view). Counterions (perchlorate) and solvent molecules are omitted for clarity.

In fact, in our hands and despite many efforts, it was not possible to either crystalize nor precipitate complexes [1a·Tb][ClO_4_]_3_ or [1a·Lu][Cl]_3_. Looking for an a work-around solution, we briefly turned our attention to other ligand structures such as pyrene macrocycle 1e, that has furnished many evidences for solution and solid state complexation with metal ions. As desired, single crystals could be formed between 1e and La(ClO_4_)_3_, by slow evaporation of a CH_3_CN solution. The resulting complex was analyzed by X-ray diffraction (Fig. 4). Globally, the structure is *C*_2_-symmetric and organized around a 1 : 1 interaction of the M^3+^ ion and the ligand structure. More precisely, a coordination mode of 10 is observed around the lanthanum ion. A bicapped square antiprismatic polyhedral is formed with the 6 oxygen atoms of the polyether ring, the two carbonyls from the amides and 2 extra water molecules.^67^ This structure for [1e·La]^3+^ fits rather well the postulated conformation in Fig. 3 for [1a·Tb]^3+^ considering nevertheless with caution the exact number of water molecules bound to the lanthanide ion that might be the result of the metal-at-play and of the solid-state geometry. In particular, we recall that the ionic radius of La(iii) is around 10% bigger than the one of Tb(iii), and therefore, La can better accommodate coordination water molecules. Moreover, Tb falls in the second half of the lanthanide transition, where smaller coordination numbers are usually reported.^68^

### Chiroptical studies (ECD and CPL)

**Fig. 5:**
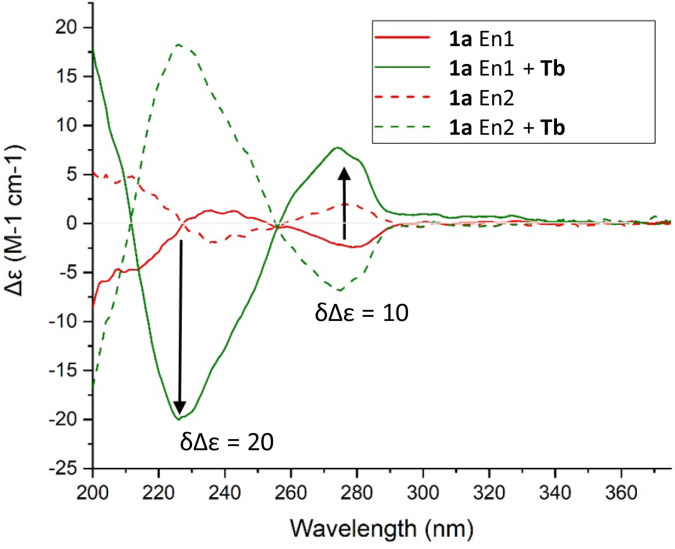
ECD spectra of 1a (red) and [1a·Tb]^3+^ (green) (solvent: acetonitrile, concentration: *ca.* 0.5 × 10^−5^ M). En1 and En2 corresponds to the 1^st^ and 2^nd^ eluted enantiomers on CHIRALPAK® IG column and a mixture of CH_2_Cl_2_–MeOH (99 : 1, 0.1% diethanolamine) as mobile phase.

Chiroptical properties of the ligand and the complex were then studied. First the ECD spectra were recorded for each single enantiomer of ligand 1a, previously separated by CSP-HPLC. As expected, the spectra presented a perfect mirror image shape and, globally, relative weak signal intensities (Fig. 5, red curves, Δ*ε* < 5 M^−1^ cm^−1^). Then, the spectra of the complexes were recorded after addition either of Tb(OTf)_3_ (Fig. 5, green curves) or of Ba(ClO_4_)_2_ (Fig. S13†).^69^ Upon the addition of the metal ions, a remarkable ECD sign reversal was observed for the two main transitions at 238 nm and 270 nm. The ECD spectra displayed mainly monosignate signals for the absorption band around 238 nm for both Tb^3+^ and Ba^2+^. Furthermore, the intensity of the ECD signals increased significantly. This (+/−)-ECD switch behavior probably originates from the conformational changes induced by the rotation of carbonyl groups (upon complexation) and the subsequent decrease of the number of conformations as computed previously on related systems.^70^ The change in intensity can be quantified using *δ*Δ*ε*, which is the difference in normalized ECD intensity in the presence and the absence of tested metal ions (eqn (1)):

1 *δ*Δ*ε* = |Δ*ε*(cation) − Δ*ε*(without)|

At the maximum absorption (225 nm), a *δ*Δ*ε* of 20 M^−1^ cm^−1^ is calculated for the terbium complex and 29 M^−1^ cm^−1^ for the barium one (235 nm). As the ECD is ligand-centered, the similarity between the spectra of [1a·Ba]^2+^ and [1a·Tb]^3+^ indicates that the two cations induce an overall similar effect onto the ligand, calling again for a similar coordination mode in the two cases for the two metals.

**Fig. 6:**
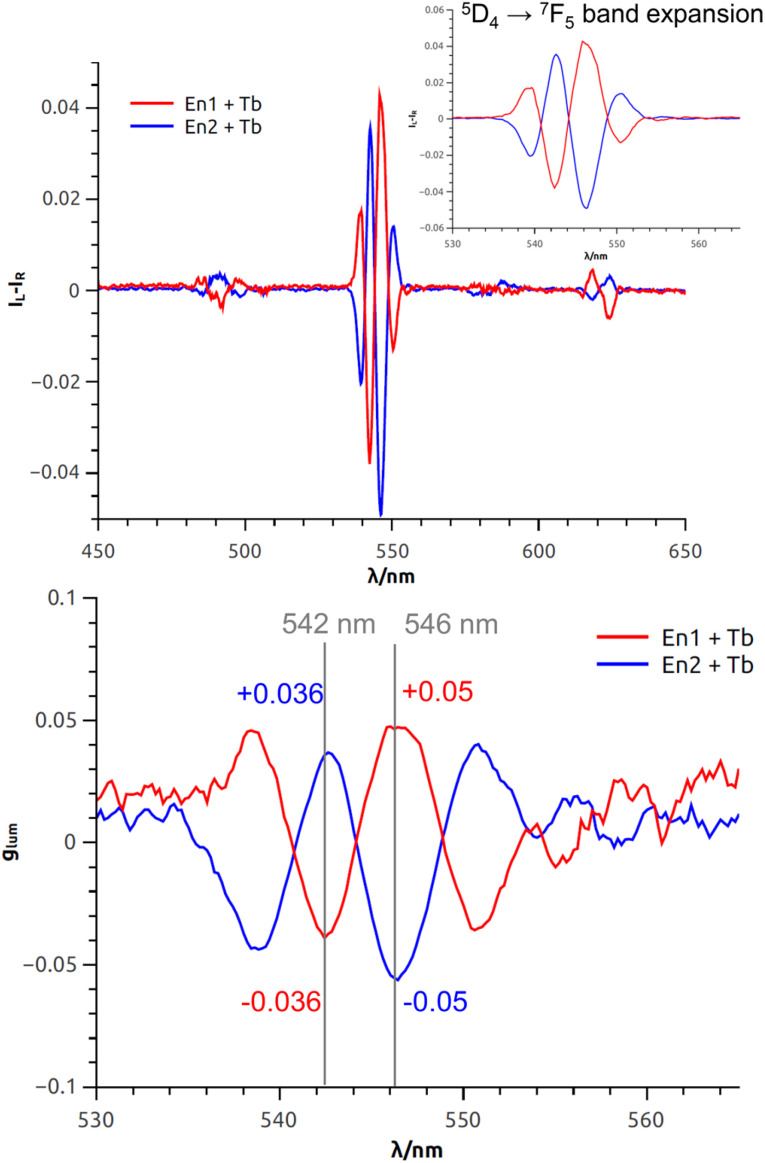
CPL spectra (top) of both enantiomers of [1a·Tb]^3+^ complexes, ^5^D_4_ → ^7^F_5_ band expansion (inset) and *g*_lum_ plot (bottom) (solvent: acetonitrile, concentration: *ca.* 2 × 10^−5^ M, *λ*_ex_: 254 nm). En1 and En2 corresponds to the 1^st^ and 2^nd^ eluted enantiomers on CHIRALPAK® IG column and a mixture of CH_2_Cl_2_–MeOH (99 : 1, 0.1% diethanolamine) as mobile phase.

Finally, but most importantly, the CPL properties of the complex [1a·Tb]^3+^ were recorded. Again, 3.0 equivalents of Tb(OTf)_3_ were added to a solution of (+) and (−)-1a in acetonitrile. The results are reported in Fig. 6. Upon excitation of the ligand (254 nm), sharp and intense CPL signals associated to ^5^D_4_ → ^7^F_J_ (J = 6 − 3) terbium transitions were recorded. The mere presence of such signal is an indication of the coordination of Tb^3+^ in a dissymmetric environment, which in this case is provided by the chiral macrocycle. In fact, the CPL signal being Tb-centered, it is possible to monitor the interaction from the point of view of the guest rather than the host (as it is the case with ECD). Thus, Tb acts as a CPL-active probe for the chirality of the macrocycle ring cavity. Such possibility is obviously precluded when Ba^2+^ or other non-luminescent cations are employed.

The fine structure observed for the ^5^D_4_ → ^7^F_5_ transition originates from the different non-degenerate M_*J*_ sublevels of the transition. To quantify the circular polarization degree of the emission, the luminescence dissymmetry factor *g*_lum_ was used as defined by eqn (2) where *I*_L_ and *I*_R_ correspond to left and right circularly polarized component of the emission respectively:2
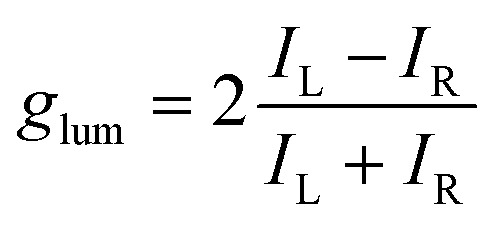


In this particular case, the *g*_lum_ factor for the ^5^D_4_ → ^7^F_5_ transition is 0.05 at 546 nm (Fig. 6, bottom). This value is in accordance with that observed for other chiral terbium complexes reported in the literature.^8,9,11,15,71–75^ As expected, the three other terbium main transitions (^5^D_4_ → ^7^F_*J*_ with *J* = 6,4,3 at 485 nm, 580 nm and 620 nm respectively) display visible but weaker CPL.

## Conclusions

In conclusion, the formation of a terbium complex with a polyether macrocyclic ligand in a 1 : 1 stoichiometry was achieved. Macrocycle 1a was readily accessed in enantiopure form (CSP HPLC) after a two-step synthesis from simple 1,4-dioxane. Upon Tb(iii) complexation, the compound exhibited a characteristic (+/−)-ECD switch behavior and an easily detectable CPL emission with a *g*_lum_ value of 0.05. In this manner, the Tb-macrocycle interaction can be observed not only from the effects on the ligand (through ECD), but also from the effects on the metal guest itself thanks to terbium CPL. The structure of the complex is also confirmed by combining data obtained from different type of analysis (^1^H-NMR analysis, spectrophotometric titrations and solid-state elucidation).

## Author contributions

A. H., F. N., A. L. T., A. F. and F. Z. performed the experiments, the characterizations and analyzed the data. C. B. performed the crystallographic measurements. A. H., F. N., A. L. T., A. F., C. B., F. Z., L. D. B. and J. L. wrote the article.

## Conflicts of interest

There are no conflicts to declare.

## Supplementary Material

DT-051-D2DT02627A-s001

DT-051-D2DT02627A-s002
