# Supplementary material for: Circularly polarized luminescence from Tb(iii) interacting with chiral polyether macrocycles
Source: Dalton Trans. 2022 Oct 4;51(43):16479–85. doi: 10.1039/d2dt02627a (PMC9641584; doi:10.1039/d2dt02627a)
Supplement: DT-051-D2DT02627A-s001 [file DT-051-D2DT02627A-s001.pdf]

Electronic Supplementary Material (ESI) for Dalton Transactions.
This journal is © The Royal Society of Chemistry 2022

Circularly polarized luminescence from Tb(III) interacting with chiral polyether macrocycles

Supporting Information

Alexandre Homberg,[a] Federica Navazio,[a,b] Antoine Le Tellier,[a] Francesco Zinna,[c] Alexandre Fürstenberg,[d,e] Céline Besnard,[f] Lorenzo Di Bari,[c] and Jérôme Lacour*[a]

[a] Department of Organic Chemistry, University of Geneva, Quai Ernest Ansermet 30,1211 Geneva 4, Switzerland. E-mail: Jerome.lacour@unige.ch.

[b] School of Science and Technology, Chemistry Division, University of Camerino, via S. Agostino n. 1, 62032 Camerino, Italy

[c] Dipartimento di Chimica e Chimica Industriale, Università di Pisa, Via Moruzzi 13, 56124 Pisa, Italy.

[d] Department of Inorganic and Analytical Chemistry, University of Geneva, 1211 Geneva, Switzerland

[e] Department of Physical Chemistry, University of Geneva, 1211 Geneva, Switzerland

[f] Laboratory of Crystallography, University of Geneva, Quai Ernest Ansermet 24, 1211 Geneva 4, Switzerland.

The dataset for this article can be found at the following DOI:

10.26037/yareta:rj5oxgsdxrhqdm7g6p6gnu4iiy

Table of Contents

1 General Information and Materials S4
1.1 CSP-HPLC S4
1.2 Optical properties S4
1.3 ^{1}H-NMR S5
2 Synthesis and characterization of organic compounds S6
2.1 Synthesis of unsaturated ester macrocycle **2** S6
2.2 Synthesis of ligands S6
2.3 Resolution of ligand **1a**[6] S7
2.4 Key chiroptical properties of ligand **1a** and complexes S8
3 Qualitative test of potential ligands S9
4 Absorbance and fluorescence spectra and titrations S10
4.1 Procedure S10
4.2 Ligand **1a** and Tb(III) S10
4.3 Titration of ligand **1a** with Tb(III) S11
4.4 Titration of ligand **1a** with Ba(II) S12
4.5 Titration of ligand **1b** with Tb(III) S12
4.6 Titration of ligand **1c** with Tb(III) S13
4.7 Titration of ligand **1d** with Tb(III) S13
5 ECD and CPL spectra S14
5.1 Procedure S14
5.2 Ligand **1a** and Tb(III) - **ECD** S15
5.3 Ligand **1a** and Ba(II) - **ECD** S16
5.4 Ligand **1a**, Tb(III) and Ba(II) - $\boldsymbol{g}_{\mathbf{abs}}$ S17
6 ^{1}H-NMR titrations S18
6.1 Procedure S18
6.2 ^{1}H-NMR spectra S18
7 Solid state structure and crystallographic data S19
7.1 Procedure S19
7.2 Data complex [**1e**·La·$(H_2O)_2$]$(ClO_4)_3$ S19
8 Luminescence lifetime measurement S21
8.1 Procedure S21

8.2 Time trace S21

9 References S22

1 General Information and Materials

1.1 CSP-HPLC

Enantiomers of ligand **1a** were resolved by chiral stationary phase HPLC on an Agilent 1260 Infinity II apparatus (quaternary pump, auto sampler, column thermostat and diode array detector) using a semi-preparative CHIRALPAK® IG column (250 x 10 mm, 5 mic). Mixtures of HPLC grade CH_2Cl_2 and MeOH (99:1, with 0.1% diethanolamine as additive) were used as mobile phase.

1.2 Optical properties

Optical properties were recorded in analytical grade solvent (acetonitrile). UV-Vis absorption spectra were recorded on a JASCO V-650 spectrophotometer at 20 °C. Electronic circular dichroism (ECD) spectra were recorded on a Jasco J-815 spectropolarimeter at 20 °C in a 1 cm cuvette.

Fluorescence spectra were measured using a Varian Cary 50 Eclipse spectrophotometer. All fluorescence spectra were corrected for the wavelength-dependent sensitivity of the detection. Fluorescence quantum yields ϕ were measured in diluted solutions (at least 5 different concentrations for each sample) with an optical density lower than 0.1 using the following equation:

$$\frac{\Phi_x}{\Phi_r} = \left(\frac{A_R(\lambda)}{A_x(\lambda)}\right)\left(\frac{n_x^2}{n_r^2}\right)\left(\frac{D_x}{D_r}\right)$$

where A is the absorbance at the excitation wavelength (λ), n the refractive index and D the integrated intensity. “r” and “x” stand for reference and sample respectively. The fluorescence quantum yields were measured in acetonitrile relative to 9,10-diphenylanthracene (ϕ = 93% in cyclohexane). Excitation of reference and sample was performed at the same wavelength.

Circularly polarized luminescence (CPL) spectra were recorded with the home-made spectrofluoropolarimeter previously described.[1] The samples were excited with a 254 nm fluorescent mercury lamp, using a 90° geometry between excitation and detection.

$Ba(ClO_4)_2$ and $Tb(OTf)_3$ salts used for titration experiments were purchased from commercial sources and used without purification.

Lifetimes were determined using the phosphorescence mode of a Fluorolog 3 spectrophotometer (Horiba Jobin Yvon) in which the lamp of the instrument is flashed. Excitation was performed at 305 nm (1 nm slit) and detection with a visible photomultiplier

tube (220-850 nm, R928P, Hamamatsu) at 545 nm (3 nm slit) at 545 nm, with an initial time gate of 50 μs.

1.3 ^{1}H-NMR

The ^{1}H NMR spectra were recorded in deuterated $CDCl_3$ using an Agilent Inova 600 (^{1}H: 600 MHz). ^{1}H NMR chemical shifts are given in ppm relative to Me_4Si using solvent resonances as internal standards (CD_3CN δ = 1.94 ppm). Data were reported as follows: chemical shift (δ) in ppm, multiplicity (s = singulet, d = doublet, t = triplet, dd = doublet of doublet, q = quartet and m = multiplet), coupling constant (Hz) and integration.

2 Synthesis and characterization of organic compounds

2.1 Synthesis of unsaturated ester macrocycle **2**

Unsaturated ester macrocycle **2** was synthetized according to previously reported procedure from the literature[2]:

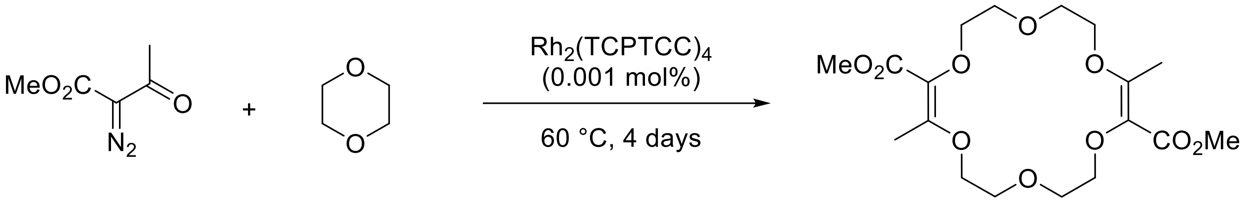

3, 140 mmol   as solvent (0.6 M)   **2**, 72%

2.2 Synthesis of ligands

Ligand **1a**,[3] **1b**,[3] **1c**,[4] **1d**,[3] **S1**,[3] **S2**[4] and **S3**[5] were synthesized according to the previously reported procedure. See Figure S1:

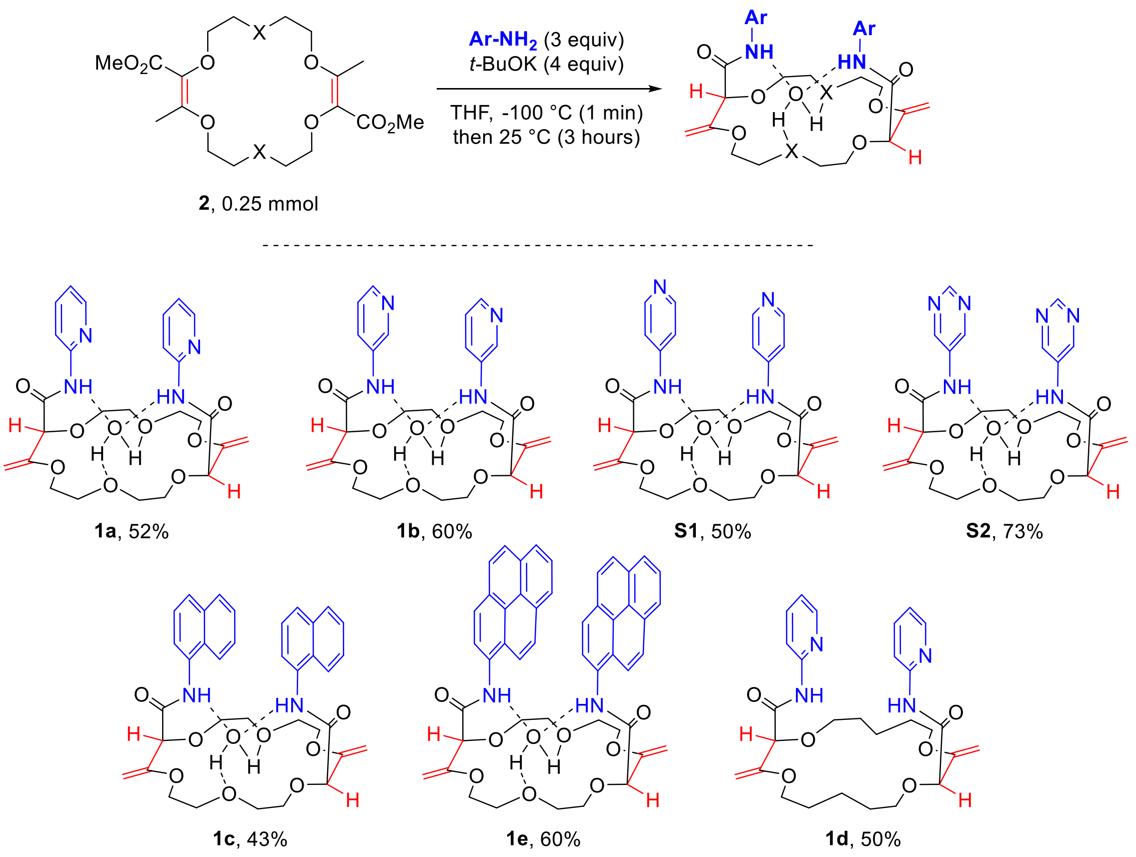

Figure S1. Synthesis of ligands.

2.3 Resolution of ligand **1a**[6]

Compound **1a** were resolved by chiral stationary phase HPLC using a semi-preparative CHIRALPAK® IG column using a mixture of CH_2Cl_2-MeOH (99:1, with 0.1% diethanolamine as additive) as mobile phase at 20 °C. It is worth mentioning that it is necessary to remove traces of diethanolamine present in the separated compounds. The residue was thus dissolved in CH_2Cl_2, the organic phase was washed three times with H_2O, dried over anhydrous Na_2SO_4, filtered and concentrated under vacuum to afford the pure products.

In the Figure **S2** is shown the HPLC traces of ligand **1a** on analytical CHIRALPAK® IG column (left, test run) and on semi preparative CHIRALPAK® IG column (right, run for resolution) with CH_2Cl_2-MeOH (99:1, 0.1% diethanolamine) as mobile phase.

analytical IG column

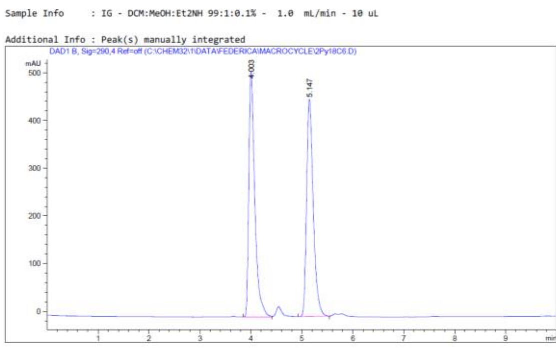

DCM : MeOH : DEA = 99 : 1 : 0.1
Inj 10 µL
Flow 1mL/min

Semi-prep IG column

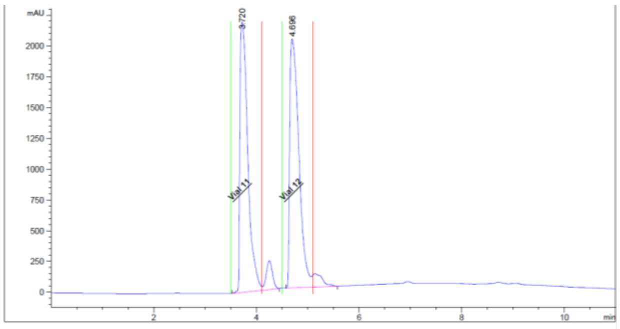

2 Vial with 10 mg
DCM : MeOH : DEA = 99 : 1 : 0.1
Inj 250 µL
Flow 5 mL/min

Figure S2. HPLC traces of the racemic mixture on analytical CHIRALPAK® IG column (left) and semi preparative (right).

In the Figure **S3** is shown the HPLC traces of ligand **1a** on analytical CHIRALPAK® IG column of the separated enantiomers: 1st eluted enantiomer on the left and 2nd eluted enantiomer on the right with CH_2Cl_2-MeOH (99:1, 0.1% diethanolamine) as mobile phase.

Analytical IG column

1st eluted enantiomer (99% *ee*)

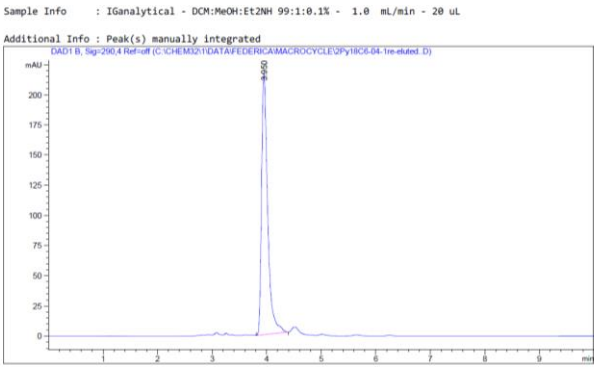

Analytical IG column

2nd eluted enantiomer (94% *ee*)

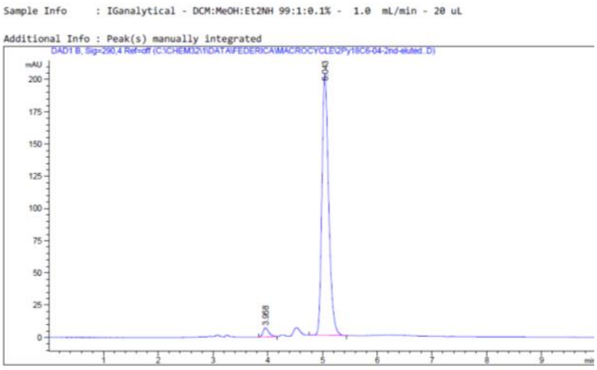

DCM : MeOH : DEA = 99 : 1 : 0.1 Inj 10 µL Flow 1mL/min

Figure S3. HPLC traces of the separated enantiomers. Left: 1st eluted enantiomer. Right: 2nd eluted enantiomer.

2.4 Key chiroptical properties of ligand 1a and complexes

ECD description for 1st eluted enantiomer of **1a** in acetonitrile, λ/nm ($\Delta\varepsilon$/$M^{-1}cm^{-1}$): 279(−2.4), 240 (+1.0).

ECD description for 1st eluted enantiomer of **1a** complexed to Tb(III) in acetonitrile, λ/nm ($\Delta\varepsilon$/$M^{-1}cm^{-1}$): 274 (+7.7), 226 (−20). *See section 5 for further precision.*

ECD description for 1st eluted enantiomer of **1a** complexed to Ba(II) in acetonitrile, λ/nm ($\Delta\varepsilon$/$M^{-1}cm^{-1}$): 274 (+4.4), 236 (−28.1). *See section 5 for further precision.*

3 Qualitative test of potential ligands

For the qualitative test, three solutions in three different vials (1 mL) were prepared and their emission was compared under UV irradiation (366 nm excitation wavelength). In the first one (reference 1), only the macrocycle of interest (<1 mg) is dissolved in acetonitrile. In the second one (reference 2), only terbium triflate (tip of a spatula) was dissolved in acetonitrile. In the third one, a mixture of the macrocycle of interest (<1 mg) and terbium triflate (tip of a spatula, excess) were dissolved in acetonitrile. In the reference 1 (1st vial), only the fluorescence of the macrocycle can be observed when visible. In the reference 2, no emission of the terbium salt was observed at this wavelength, but for the third vial (macrocycle/Tb mixture) resulted in the characteristic green terbium emission (see below). Combination of ligand **1a** and terbium presents the most efficient luminescence and were selected for this study.

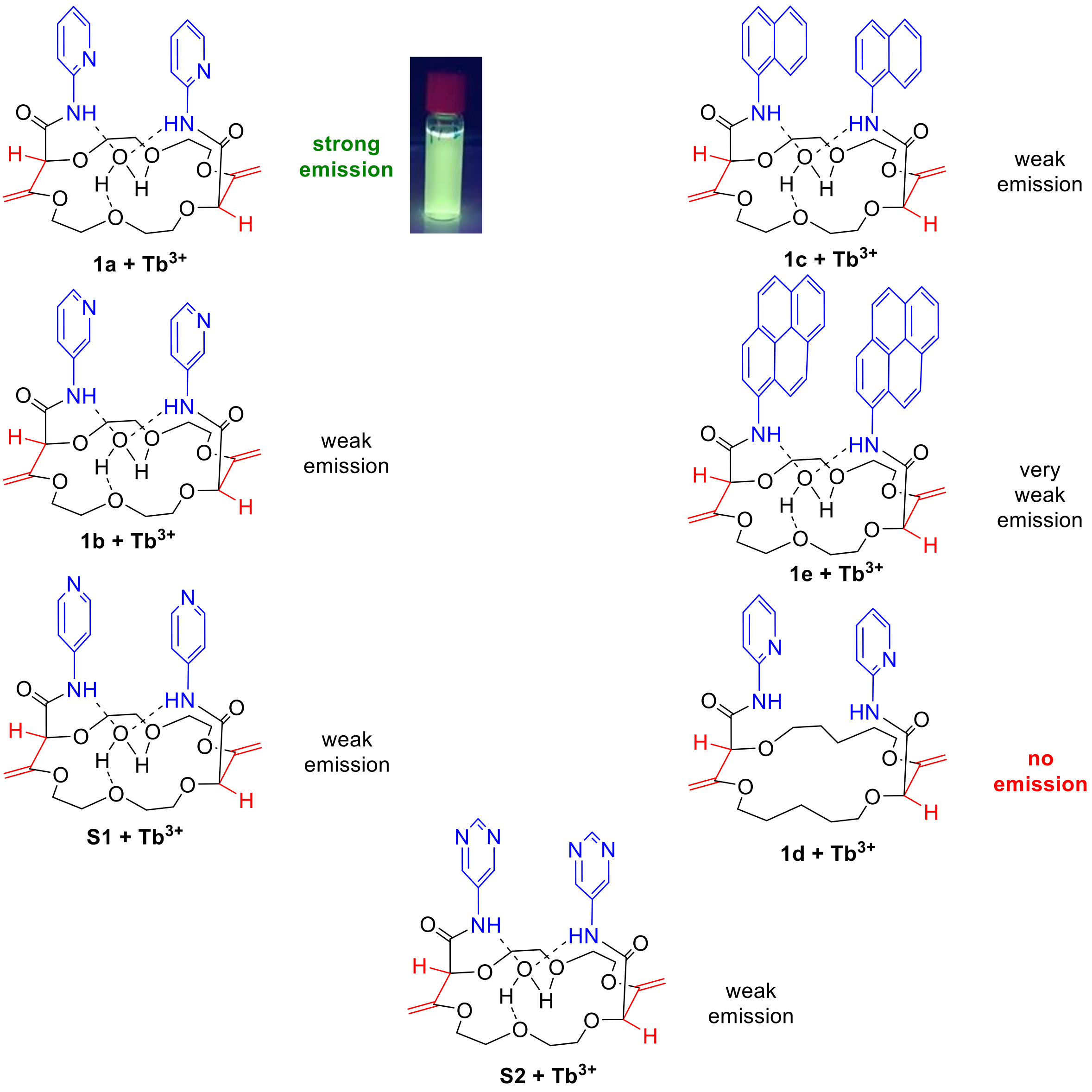

Figure S4. Ligand tested with terbium(III) and qualitative results of the mixture (picture under 366 nm irradiation).

4 Absorbance and fluorescence spectra and titrations

4.1 Procedure

In a typical experiment, UV-Vis absorbance and fluorescence spectra of a solution of interest compound (ca. 0.5·10^{-6} M) in acetonitrile were recorded in a 1 cm cell. For the complexation experiments, an excess of $Tb(OTf)_3$ (or $Ba(ClO_4)_2$) or an aliquot of a $Tb(OTf)_3$ (or $Ba(ClO_4)_2$) solution in acetonitrile (ca. 2·10^{-3} M) was added to the solution and the UV-Vis absorbance and fluorescence spectra were recorded again.

4.2 Ligand **1a** and Tb(III)

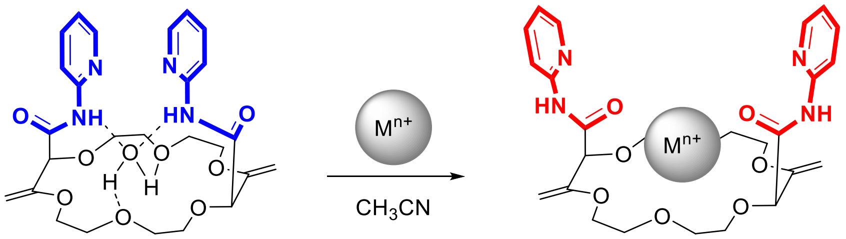

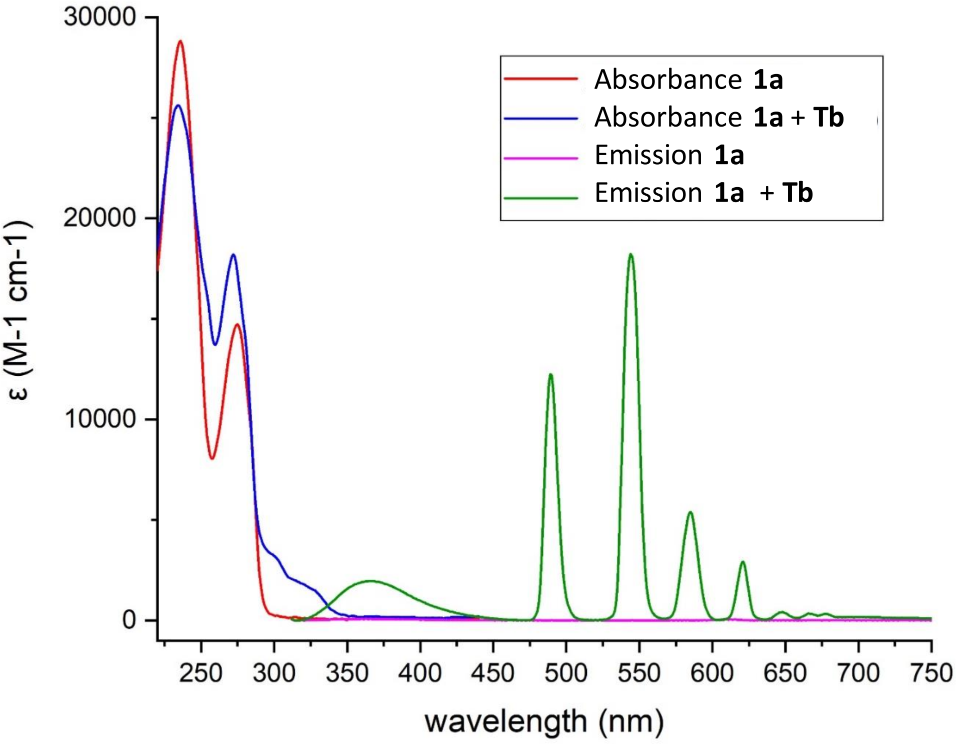

Figure S5. Absorbance (red and blue lines) and fluorescence (pink and green lines) spectra of ligand **1a** without (red and pink lines) or with 3.0 equivalents of Tb(III) (blue and green lines).

4.3 Titration of ligand **1a** with Tb(III)

In a typical experiment, a known aliquot of a $Tb(OTf)_3$ solution in acetonitrile (ca. $2{\cdot}10^{-3}$ M) was added to a solution of the ligand (ca. $0.5{\cdot}10^{-6}$ M) in acetonitrile. Spectra were recorded from 0 equivalent of Tb(III) to 4.0 equivalents.

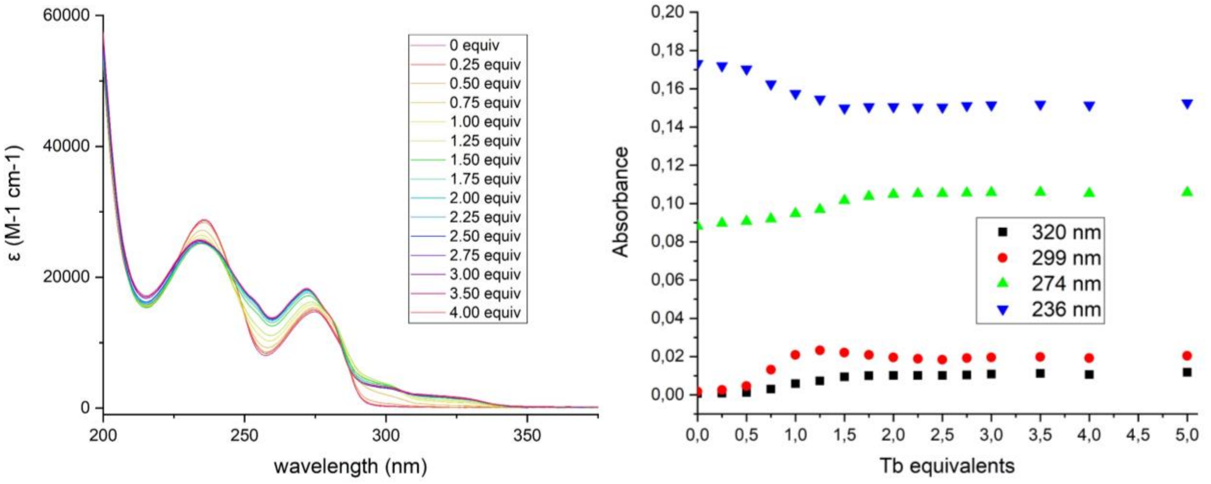

Figure S6. Titrations spectra in absorbance of ligand **1a** with Tb(III): 0 to 4.0 equivalents. Left: absorbance spectra. Right: evolution of absorbance as function of the equivalents added at different relevant wavelength.

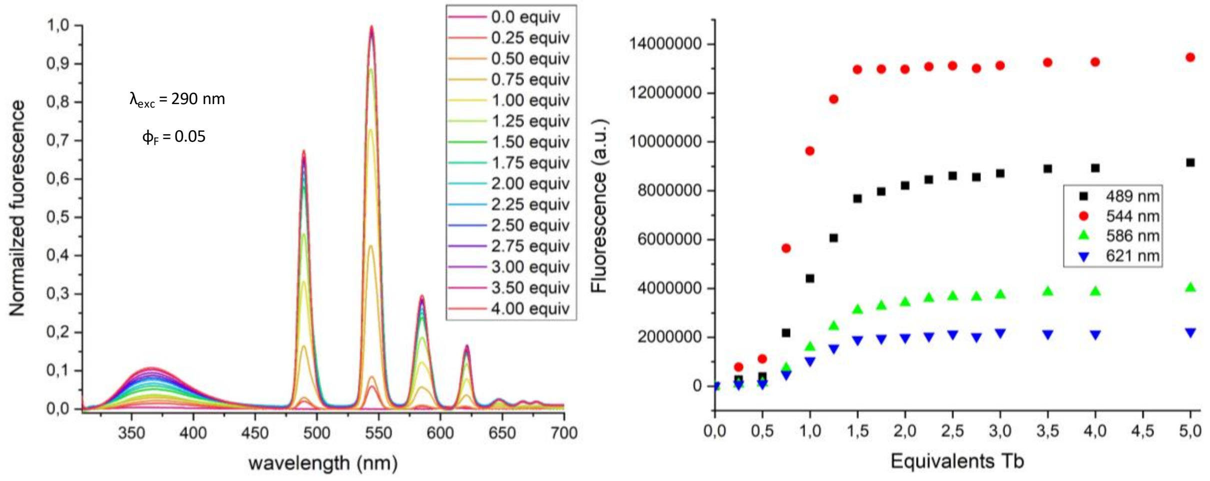

Figure S7. Titrations spectra in emission of ligand **1a** with Tb(III): 0 to 4.0 equivalents. Left: normalized fluorescence spectra. Right: evolution of fluorescence as function of the equivalents added at different relevant wavelength.

4.4 Titration of ligand **1a** with Ba(II)

In a typical experiment, a known aliquot of a $Ba(ClO_4)_2$ solution in acetonitrile (ca. $4{\cdot}10^{-3}$ M) was added to a solution of the ligand (ca. $0.5{\cdot}10^{-6}$ M) in acetonitrile. Spectra were recorded from 0 equivalent of Ba(II) to 4.0 equivalents.

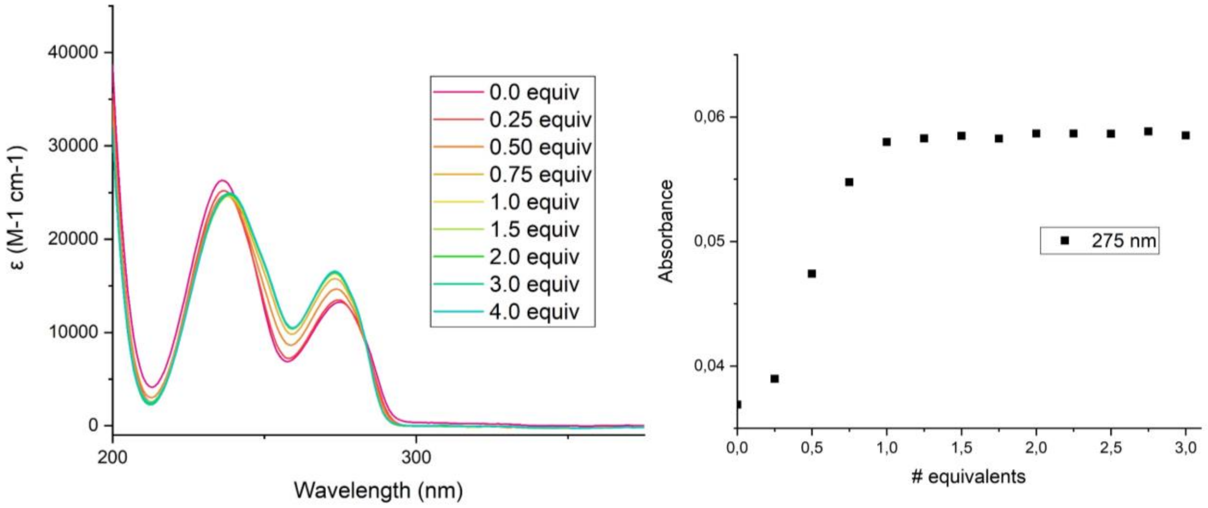

Figure S8. Titrations spectra in absorbance of ligand **1a** with Ba(II): 0 to 4.0 equivalents. Left: absorbance spectra. Right: evolution of absorbance as function of the equivalents added at 274 nm.

4.5 Titration of ligand **1b** with Tb(III)

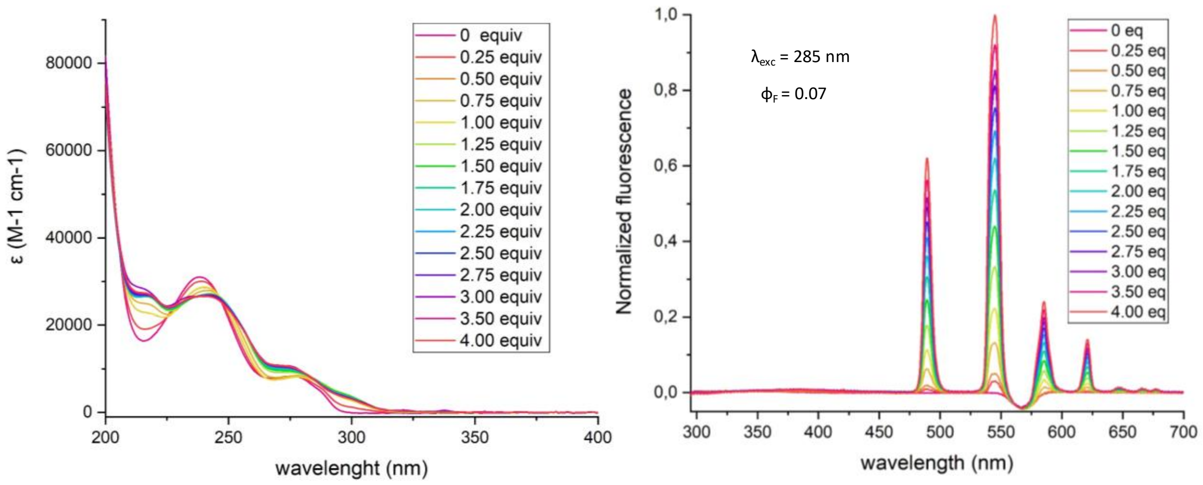

Figure S9. Titrations spectra of ligand **1b** with Tb(III): 0 to 4.0 equivalents. Left: absorbance spectra. Right: normalized fluorescence spectra.

4.6 Titration of ligand **1c** with Tb(III)

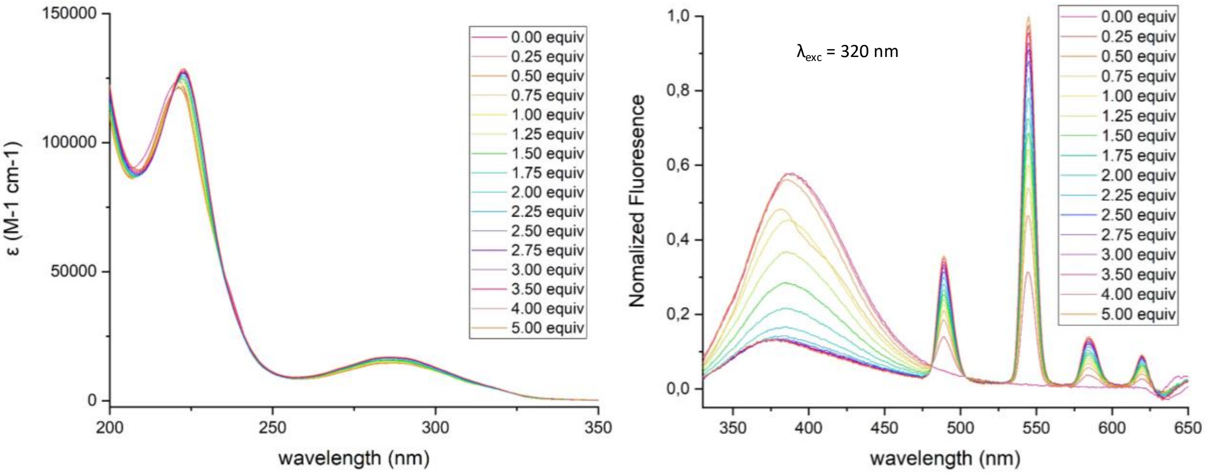

Figure S10. Titrations spectra of ligand **1c** with Tb(III): 0 to 5.0 equivalents. Left: absorbance spectra. Right: normalized fluorescence spectra.

4.7 Titration of ligand **1d** with Tb(III)

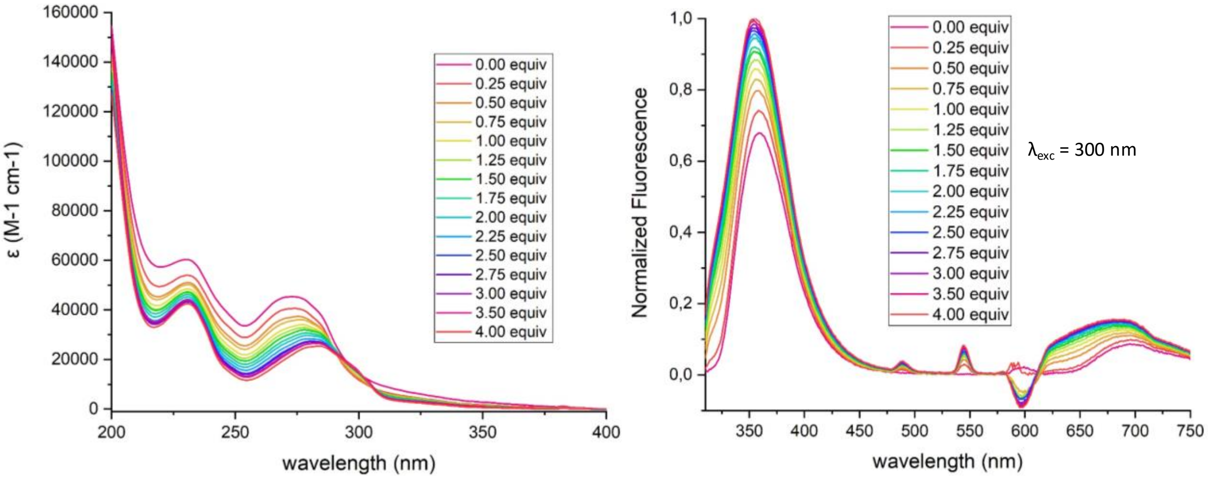

Figure S11. Titrations spectra of ligand **1d** with Tb(III): 0 to 4.0 equivalents. Left: absorbance spectra. Right: normalized fluorescence spectra.

5 ECD and CPL spectra

5.1 Procedure

For **ECD**:

In a typical experiment, the ECD spectrum of a solution of enantiopure ligand (ca. $0.5 \cdot 10^{-5}$ M) in acetonitrile was recorded in a 1 cm cell at 20 °C. For the complexation experiments, 3.0 equivalents of $Tb(OTf)_3$ or $Ba(ClO_4)_2$ (ca. $2 \cdot 10^{-3}$ M stock solutions in acetonitrile) were added to the ligand solution and the ECD spectrum was recorded again.

The change in intensity in ECD is quantified using δΔε, which is the difference in normalized ECD intensity in presence and absence of tested metal ions:

$$\delta\Delta\varepsilon = |\,\Delta\varepsilon(\text{cation}) - \Delta\varepsilon(\text{without})| \quad \text{(S1)}$$

For **CPL**:

The CPL spectrum of a solution containing the enantiopure ligand (ca. $2 \cdot 10^{-5}$ M) in acetonitrile and 3.0 equivalents of $Tb(OTf)_3$ (from a ca. 10^{-3} M stock solution in acetonitrile) was recorded in a 1 cm cell.

The circular polarization degree of the emission is quantifying using the luminescence dissymmetry factor g_{lum} defined by equation S2 where I_L and I_R correspond to left and right circularly polarized component of the emission respectively:

$$g_{lum} = 2\,\frac{I_L - I_R}{I_L + I_R} \quad \text{(S2)}$$

5.2 Ligand **1a** and Tb(III) - **ECD**

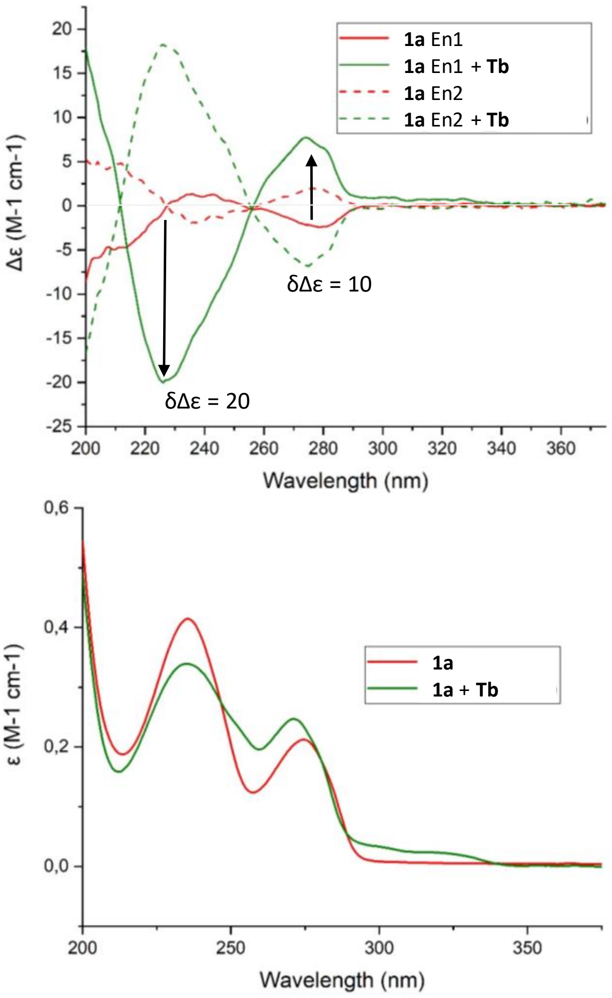

Figure S12. ECD (top) and absorbance (bottom) spectra ligand **1a** (red) and [**1a**·Tb]$^{3+}$ complex (green). En1 and En2 corresponds to the 1st and 2nd eluted enantiomers on CHIRALPAK® IG column and a mixture of CH_2Cl_2-MeOH (99:1, 0.1% diethanolamine) as mobile phase.

5.3 Ligand **1a** and Ba(II) - **ECD**

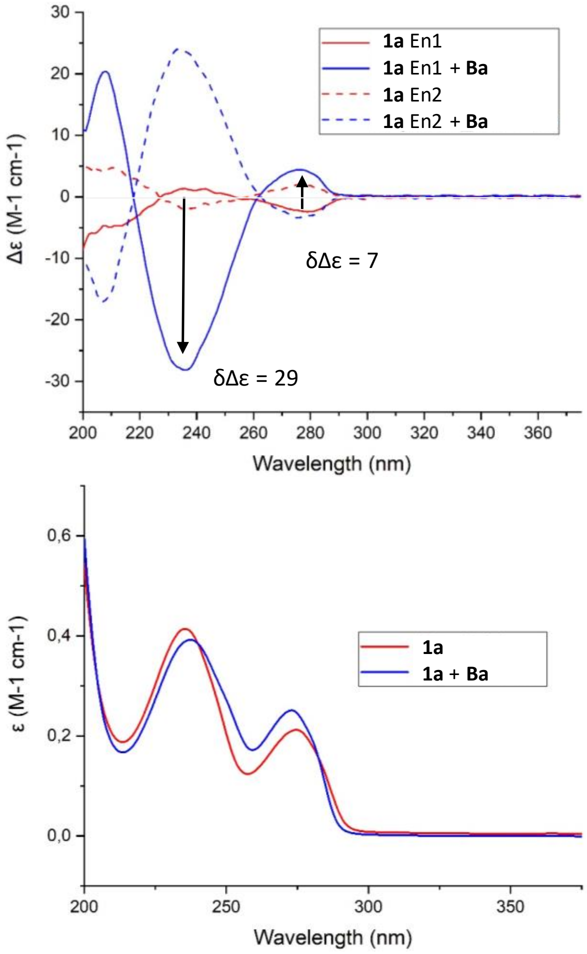

Figure S13. ECD (top) and absorbance (bottom) spectra ligand **1a** (red) and [**1a**·Ba]$^{2+}$ complex (blue). En1 and En2 corresponds to the 1st and 2nd eluted enantiomers on CHIRALPAK® IG column and a mixture of CH_2Cl_2-MeOH (99:1, 0.1% diethanolamine) as mobile phase.

5.4 Ligand **1a**, Tb(III) and Ba(II) - g_{abs}

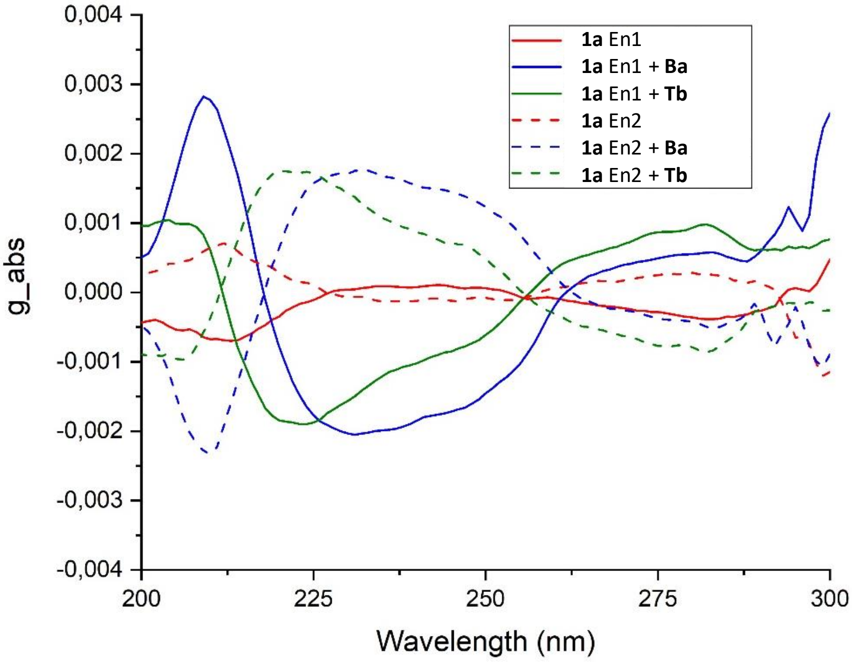

Figure S14. g_{abs} spectra of ligand **1a** (red), [**1a**·Tb]$^{3+}$ (green) and [**1a**·Ba]$^{2+}$ (blue) complexes. En1 and En2 corresponds to the 1st and 2nd eluted enantiomers on CHIRALPAK® IG column and a mixture of CH_2Cl_2-MeOH (99:1, 0.1% diethanolamine) as mobile phase.

6 ^{1}H-NMR titrations

6.1 Procedure

To an NMR tube, $LuCl_3$ was added (0, 0.5, 1 and 2 equivalents) as a solid. Just before the measurement 0.5 mL of a 15 mM solution of **1a** was added and the tube was shaken, and the ^{1}H spectrum was recorded.

6.2 ^{1}H-NMR spectra

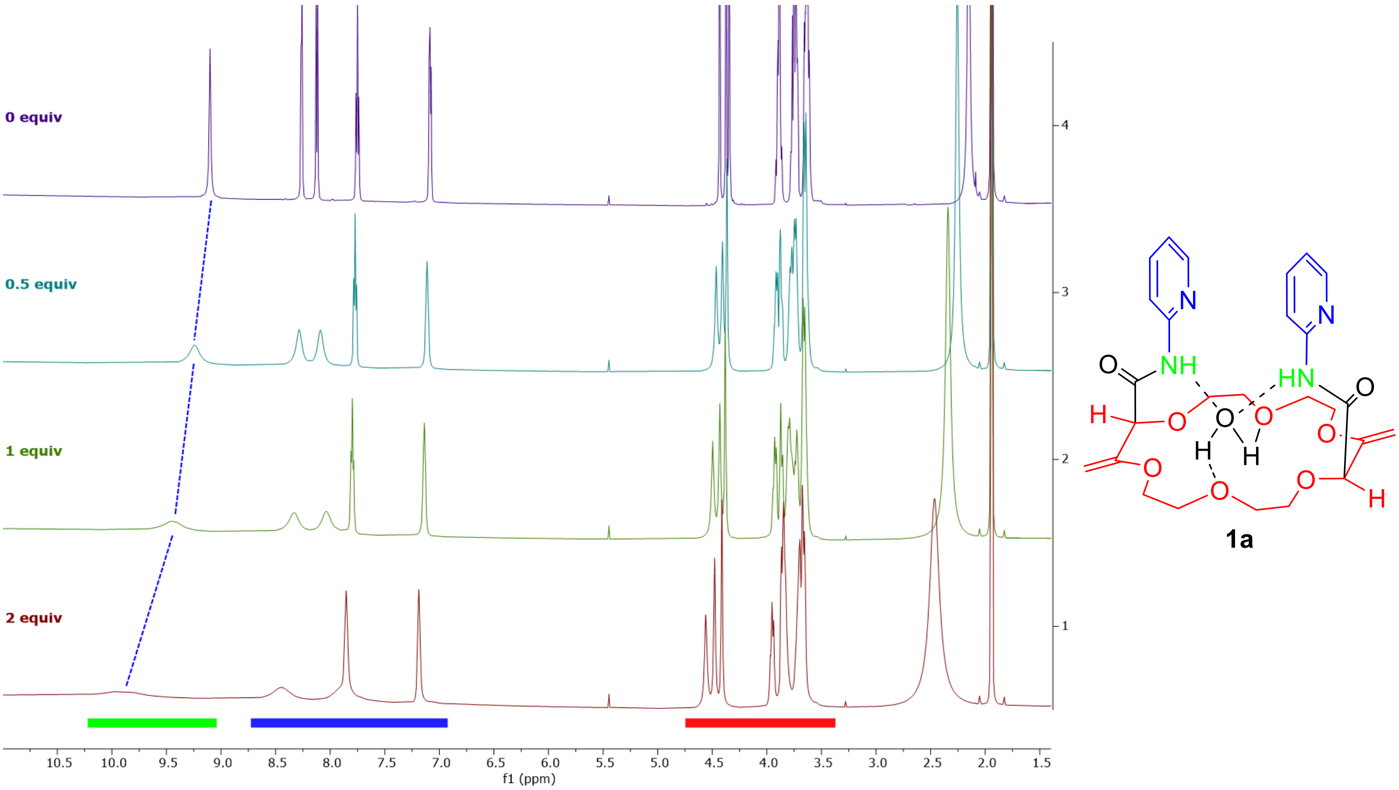

Figure S15, ^{1}H NMR titration of **1a** with $LuCl_3$ (0, 0.5, 1, 2 equivalents) c = 15 mM, 600 MHz

7 Solid state structure and crystallographic data

7.1 Procedure

About 5 mg of **1a** were dissolved in 2 mL of MeCN and a large excess of $La(ClO_4)_3$ was added. The solution was filtered and the solvent allowed to slowly evaporate at room temperature over the course of one week.

7.2 Data complex [**1e**·La·$(H_2O)_2$]$(ClO_4)_3$

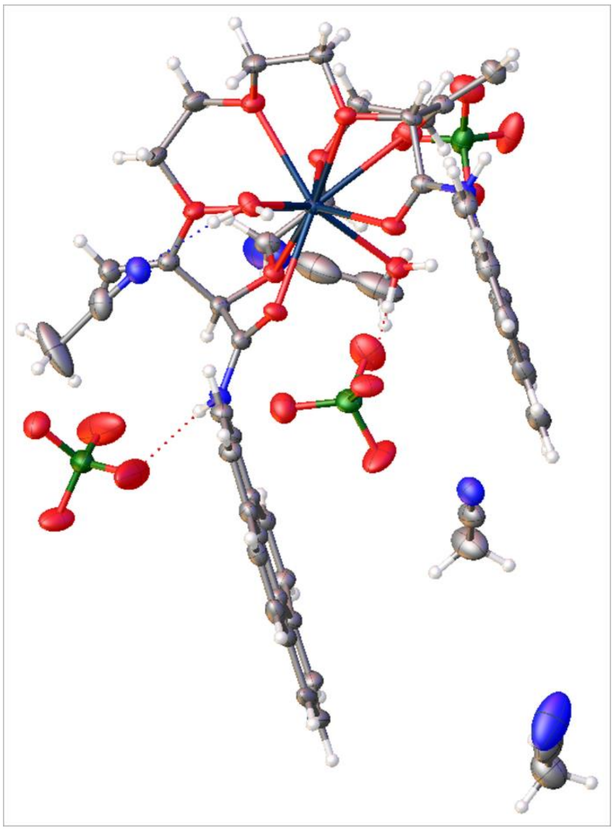

Figure S16. Crystal data and structure refinement for [**1e**·La·$(H_2O)_2$]$(ClO_4)_3$

CCDC number	2189306
Empirical formula	C56 H58 Cl3 La N6 O22
Formula weight	1412.34
Temperature	120.00(11) K
Crystal system	Monoclinic
Space group	P21/c
Unit cell dimensions	a = 23.5523(2) Å α = 90°
	b = 11.12589(12) Å β = 99.5672(10) °
	c = 23.3538(2) Å γ = 90°
Volume	6034.52(11) Å^3
Z	4
Density (calculated)	1.555 g/cm^3
F(000)	2880.0
Crystal size	0.591 × 0.136 × 0.036 mm^3
2Θ range for data collection	7.614 to 149.28°
Index ranges	-29 ≤ h ≤ 29, -13 ≤ k ≤ 13, -29 ≤ l ≤ 29
Reflections collected	19051
Independent reflections	19051 [Rint = ?, Rsigma = 0.0100]
Data/restraints/parameters	19051/3/809
Goodness-of-fit on F^2	1.034
Final R indexes [I>=2σ (I)]	R1 = 0.0439, wR2 = 0.1169
Final R indexes [all data] R	1 = 0.0465, wR2 = 0.1192
Largest diff. peak/hole	1.19/-1.26 e Å^{-3}

8 Luminescence lifetime measurement

8.1 Procedure

A 10^{-5} M solution of **1a** (S1) and a 10^{-3} M solution of $Tb(OTf)_3$ (S2) both in MeCN or MeCN-d3 were prepared. The samples were prepared by mixing the 2 solutions directly in a cuvette.

The lifetime of complex **1a** in acetonitrile was determined using the phosphorescence mode of a Fluorolog 3 spectrophotometer (Horiba Jobin Yvon) in which the lamp of the instrument is flashed. Excitation was performed at 305 nm (1 nm slit) and detection with a visible photomultiplier tube (220-850 nm, R928P, Hamamatsu) at 545 nm (3 nm slit) at 545 nm, with an initial time gate of 50 μs.

8.2 Time trace

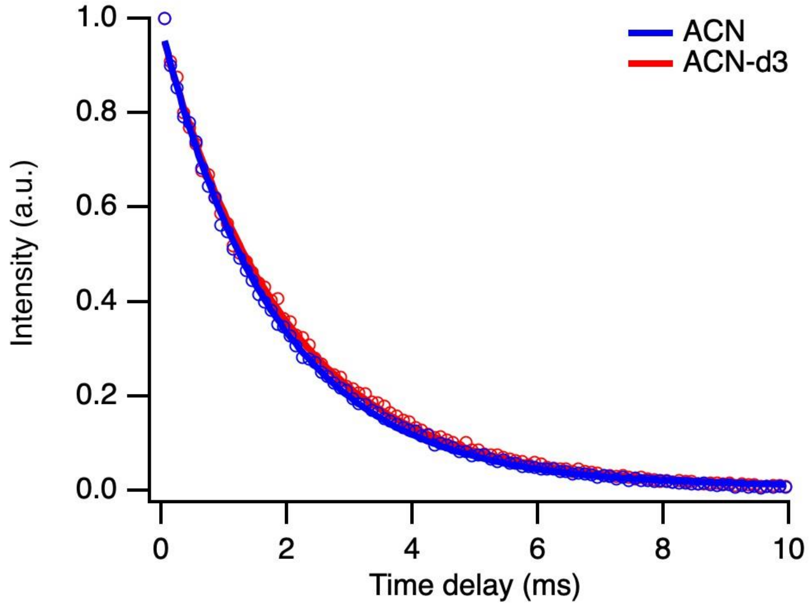

Figure S17. Luminescence decay of [**1a**·Tb][ClO_4]$_3$ at 545 nm upon 305 nm excitation in MeCN and MeCN-d3 solutions. Solid lines are exponential fits to the data points (open circles).

9 References

1) F. Zinna, T. Bruhn, C. A. Guido, J. Ahrens, M. Bröring, L. Di Bari and G. Pescitelli, *Chem. Eur. J.*, 2016, **22**, 16089-16098.
2) D. Poggiali, A. Homberg, T. Lathion, C. Piguet and J. Lacour, *ACS Catal.*, 2016, **6**, 4877-4881.
3) M. Vishe, R. Hrdina, A. I. Poblador-Bahamonde, C. Besnard, L. Guénée, T. Bürgi and J. Lacour, *Chem. Sci.*, 2015, **6**, 4923-4928.
4) E. Brun, K.-F. Zhang, L. Guénée and J. Lacour, *Org. Biomol. Chem.*, 2020, **18**, 250-254.
5) Z. Jarolímová, M. Vishe, J. Lacour and E. Bakker, *Chem. Sci.*, 2016, **7**, 525-533.
6) We acknowledge the help of Dr Pilar Franco and Mrs Assunta Green (Chiral Technologies, Illkirch, France) for their help and advices for the initial screening of columns and solvent conditions.
